# Dilemma of women’s passive smoking

**DOI:** 10.4103/1817-1737.78410

**Published:** 2011

**Authors:** Randa M Mostafa

**Affiliations:** *Department of Basic Medical Sciences College Of Medicine, Sharjah University, Sharjah, President of Emirates Menopause Society, UAE*

Tobacco smoking has been and still primarily a custom and an addiction of men, leaving women and children as the majority of the world’s passive or involuntary smokers.[1] The problem of smoking at home is particularly difficult for women in many cultures especially Arab cultures where it may not be acceptable for a woman to ask her husband not to smoke at home or in the presence of her children.

As more than 4000 chemicals have been identified in tobacco smoke, at least 250 of which are known to be harmful and more than 50 of which are known to cause cancer.[2] People in places that allow smoking can be subject to significant levels of toxins, as pollution from tobacco smoke can reach levels that are much higher than levels of other environmental toxins, such as particles found in automobile exhaust. Studies has shown that pollution levels in door places are higher than levels found on busy roadways, in closed motor garages, and during fire storms.[3] Furthermore, smoke can spread from one room to another, even if doors to the smoking area are closed. Singer and his colleagues state that toxic chemicals from tobacco smoke contamination persist well beyond the period of active smoking, and then cling to rugs, curtains, clothes, food, furniture, and other materials. These toxins can remain in a room weeks and months after someone has smoked there[4] even if windows are opened or fans or filters are used. Filters can become a source for deposited chemicals that are then recycled back into the air of a room rather than removed. Tobacco toxins that build up overtime, coating the surfaces of room elements and materials and smoker’s belongings are sometimes referred to as third hand smoking.[5] Hence, passive smoking is still a women’s important issue because of its negative impact on the health of women and her children.

Hull *et al*. concluded that passive smoking of women are associated with delayed conception.[6] Pregnancy represents a period of particular vulnerability, during which exposure to tobacco smoke may adversely affect the developing fetus. Maternal smoking during pregnancy is known to be associated with adverse pregnancy outcomes, including low birth weight, intrauterine growth retardation, premature delivery, spontaneous abortion, placental abruption, placenta praevia, perinatal mortality, and ectopic pregnancy, especially in older mothers. There is also increased postnatal morbidity and mortality relating to deficits in pulmonary function and neurocognitive development.[7] These effects are proportionate to dose, starting with passive smoke.[7,8] Furthermore, it seems that the exposure of the pregnant woman to passive smoking by her partner is also detrimental, as it results in a significant passage of the metabolites of nicotine through the placenta.[8] In addition, nicotine may be less rapidly metabolized in pregnant women.[9] For children, the effect of exposure to environmental tobacco smoke ETS also varies from infancy through childhood and adolescence. Infancy and childhood represent periods of vulnerability because of an immature defense mechanism and because organs such as the lungs are still growing. Early childhood increases the risk of severe lower respiratory illnesses such as bronchitis and pneumonia.[1] There are various hypotheses for the biological action of smoking that leads to earlier menopause. For example, women who smoke present lower levels of estradiol in the middle of the cycle and in the luteal phase, in comparison with nonsmokers. Experimental models using animals have suggested that nicotine increases the loss of follicles in the ovary and blocks the enzyme aromatase, which is responsible for converting androgens into estrogens. Furthermore, these models have demonstrated that the peak level of luteinizing hormone in the middle of the cycle is delayed or nonexistent.[10] Diminished ovarian reserves are more common among women who smoke than among those who do not, which would at least partially explain why infertility is more common among smokers.[11] Smoking has also been associated with increased use of the 2-hydroxylation pathway for estradiol metabolism in the liver. This produces increased levels of 2-hydroxyestrogen, which almost totally lacks peripheral activity.[12] Smoking also seems to increase the quantity of androgens produced by the suprarenal glands, which contributes towards an antiestrogen effect.[13] Polyaromatic hydrocarbons present in cigarettes may induce the microsomal cytochrome P-450 (which metabolizes steroidal hormones) to increase the formation of catechol metabolites of estradiol, which are weaker estrogens and therefore less capable of producing the beneficial effects of this hormone.[9] An association has also been found between the preapoptotic protein Bax and the hydrocarbons present in cigarettes, which suggest that exposure to these aromatic hydrocarbons, could induce the expression of Bax in oocytes. This would cause apoptosis and thus lead to earlier ovarian failure.[9,14]

On conclusion, in addition to all national tobacco policies of implementing extensive restrictions on smoking in public places and work places, empowering women to limit exposure to environmental tobacco smoke ETS at least in their home is a challenge to public health policy makers because it addresses gender inequality in the private sphere.
